# Nutrition education linked to agricultural interventions improved child dietary diversity in rural Cambodia

**DOI:** 10.1017/S0007114516003433

**Published:** 2016-10-05

**Authors:** Anika Reinbott, Anna Schelling, Judith Kuchenbecker, Theresa Jeremias, Iean Russell, Ou Kevanna, Michael B. Krawinkel, Irmgard Jordan

**Affiliations:** 1 Justus Liebig University Giessen, Wilhelmstr 20, 35392 Giessen, Germany; 2 Food and Agriculture Organization of the United Nations, Nutrition Education and Consumer Awareness Group (ESNE), Nutrition Division (ESN), Viale delle Terme di Caracalla, 00153, Rome, Italy; 3 Food and Agriculture Organization of the United Nations (FAO), No. 5, Street 370, Boeung Keng Kang I, 12302 Phnom Penh, Cambodia; 4 National Maternal and Child Health Center, No. 31A, Rue de France (Street 47), 12202 Phnom Penh, Cambodia

**Keywords:** Community-based nutrition, Nutrition education, Child dietary diversity, Stunting

## Abstract

Poor infant and young child feeding (IYCF) practices are major determinants of chronic malnutrition. The main objective of this study was to assess the impact of a nutrition education (NE) programme aimed at promoting improved IYCF behaviours in combination with an agriculture intervention on children’s dietary diversity and nutritional status. From 2012 to 2014, a cluster randomised trial was rolled out in Cambodia in the context of an agriculture and nutrition project of the FAO of the UN. The cross-sectional baseline study was carried out in sixteen pre-selected communes in 2012. Restricted randomisation allotted the communes to either intervention (NE and agriculture intervention) or comparison arms (agriculture intervention only). The impact survey was conducted as a census in all FAO project villages in 2014. Caregivers of children aged 0–23 months were interviewed using standardised questions on socio-economic status and dietary diversity (24-h recall). Anthropometric measurements were taken. A difference-in-differences model was applied. The sample comprised 743 households with children ≥6 months of age at baseline and 921 at impact. After 1 year of NE, 69 % of the intervention households reported to have participated in the NE. Estimated mean child dietary diversity was significantly different at impact between comparison and intervention (3·6 and 3·9, respectively). In particular, the consumption of pro-vitamin A-rich foods and other fruits and vegetables increased. No treatment effects on height-for-age *Z*-scores could be shown. NE led to improvements in children’s diets. For effects on growth, it is assumed that longer NE activities are required to achieve sustainable behaviour change of age-appropriate infant feeding.

Poor knowledge of infant and young child feeding (IYCF) in addition to household food insecurity is a major determinant of chronic malnutrition among children aged 6–23 months. In this age group, often referred to as the ‘critical window’, the timeliness of the introduction, quality, quantity and appropriateness of complementary food are crucial to ensure adequate growth as well as motor and mental development^(^ ^1^ ^,^ ^2^ ^)^. However, to date, the prevalence of chronic malnutrition reflected in stunted growth globally remains high, with 162 million children under 5 years of age being affected^(^ ^3^ ^)^. Risk factors to be addressed vary by country and context but have the challenge of appropriate complementary feeding practices in common^(^ ^1^ ^,^ ^4^ ^)^. Community-based nutrition education interventions have the potential to improve complementary feeding practices by increasing the knowledge of age-appropriate diets as well as caring and feeding practices. Through raised awareness and knowledge, changes in behaviour can be expected, and with improved quality of infants’ diets adequate growth could be expected^(^ ^4^ ^,^ ^5^ ^)^. Even in food-secure populations, lack of knowledge of appropriate IYCF practices may lead to inadequate nutritional intakes, and thus negatively impact on infants’ health and development^(^ ^5^ ^)^.

Food insecurity can be a major constraint for caregivers to make use of gained IYCF knowledge, as the availability, affordability and utilisation of food in a household are directly linked to the diets of young children^(^ ^6^ ^,^ ^7^ ^)^. Combined nutrition education and agricultural interventions address not only poor IYCF knowledge and practices but also household food insecurity. Globally, the number of such programmes has increased in recent years, but little is known about the evidence for the effects of the approach.

Stunting prevalence (height-for-age *Z*-score (HAZ) ≤−2 sd) among children aged 0–23 months in Cambodia was 22 % in 2014, showing a slight decrease in comparison with 2010, when 26 % of all children under 2 years of age were stunted^(^ ^8^ ^,^ ^9^ ^)^. The demand for options for sustainably improving IYCF practices to further reduce stunting prevalence has been addressed by a number of programmes in Cambodia, but evidence for the determination of best practices remains limited.

Hence, the main objective of this study was to assess the impact of a nutrition education programme that aimed at improving IYCF practices by combining agricultural interventions with training on child feeding. A unique aspect of this study was the combination of the implementation of nutrition education interventions under the responsibility of the FAO of the UN and the research performed by an independent research team from Justus Liebig University Giessen, Germany.

## Methods

**Fig. 1:**
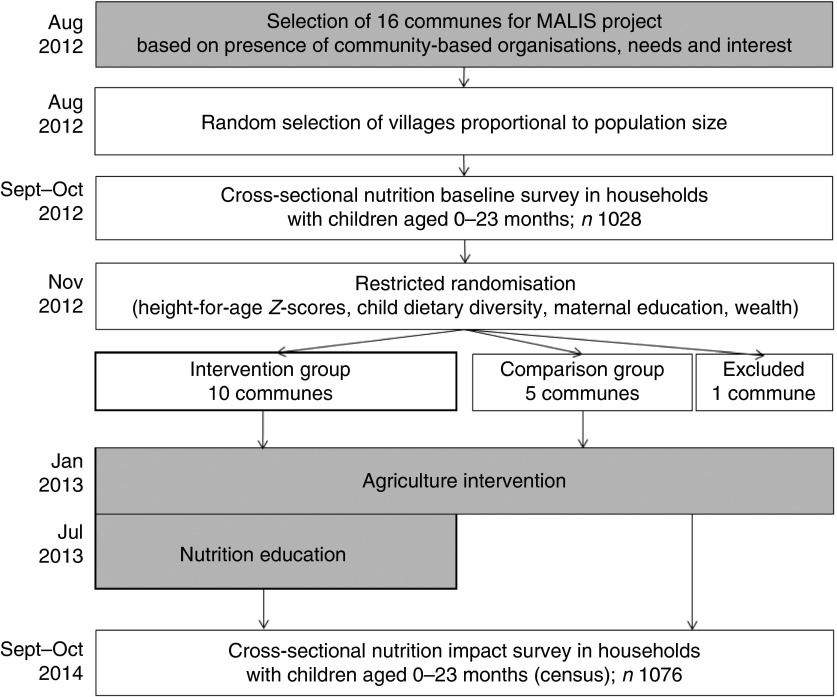
Research design. 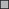, FAO project components; 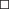, Liebig University research activities. MALIS, improving market linkages for smallholder farmers.

From 2012 to 2014, a cluster randomised controlled trial was rolled out in Preah Vihear and Oddar Meanchey, provinces in Northern Cambodia, in the context of a FAO food security and nutrition project. The FAO project ‘Improving market linkages for smallholder farmers’ (MALIS) included a nutrition education programme to improve IYCF practices linked with the promotion of improved farming systems and building up market linkages. Working closely with the respective provincial departments of the Ministries of Agriculture, Forestry and Fisheries, Women’s Affairs and Health, as well as with non-governmental organisations (NGO), MALIS project selected a total of sixteen communes in Preah Vihear and Oddar Meanchey in August 2012. The selection was based on the presence of community-based organisations, farmers’ needs and interest. FAO conducted training of trainers in conjunction with government staff before any field activities commenced. The agriculture component comprised farmer field schools, farmer business schools and the provision of input credit, mainly through agricultural fairs. The nutrition education programme commenced in August 2013 in villages where farmer field and farmer business schools had already been rolled out by the MALIS project and which the research team had identified as intervention villages (Fig. 1). For the nutrition education programme, caregivers with a child aged 5–18 months were recruited on the basis of their interest in participating; priority was given to caregiver–child pairs from households already participating in a farmer field or farmer business school.

The study was approved by the Institutional Review Board of Justus Liebig University and the National Ethics Committee for Health Research in Cambodia, and is registered at the German Clinical Trials Register (no. DRKS00004379).

### Baseline survey

A baseline survey was carried out by the independent research team in all sixteen communes targeted by the MALIS project in six districts of Preah Vihear and Oddar Meanchey provinces in September/October 2012. About 17 650 eligible MALIS beneficiaries lived in the area. Only farm households with children aged 0–23 months were enrolled in the survey. Other inclusion criteria were being a resident in the sampled area, being randomly selected and being willing to participate. Informed consent was obtained from each caregiver before data collection.

Sample size for the baseline survey was determined using Emergency Nutrition Assessment for smart sample size calculator^(^ ^10^ ^)^. The aim was to assess mean height-for-age of children aged 0–23 months and determinants of stunting at the commune level to facilitate balancing intervention and comparison areas. A total population of 15 000 children <2 years of age was considered as living in the survey area. The estimated stunting prevalence of 50 %, a desired precision of ±5 % and a design effect of 3 led to the calculated sample size of 1124 children.

The sampling was conducted using a two-stage probability sampling strategy. Initially, three villages per commune were sampled proportional to population size. At the second sampling stage, twenty-three households with children aged 0–23 months were randomly selected in each village, where more than twenty-three children in this age range lived in the selected village. If there were exactly twenty-three children, all caregiver–child pairs were invited to participate. If the village had less than twenty-three eligible children, households were selected at random from the nearest adjacent village to complete the required sample size. Children with missing birth certificates, vaccination cards or where the month of birth of the child could not be estimated and/or the primary caregiver was not available were excluded from the study (*n* 88). A total of 1032 households could finally be interviewed. Out of 1032 data sets, one child was above 23 months of age and was thus excluded, and three children were excluded as they were twins with the child code 2. In total, 1028 households with a child between 0 and 731 d from forty-nine villages were eligible for data collection and analysis.

### Randomisation of intervention and comparison clusters

Intervention and comparison areas were identified using the software package ‘Experiment’ and the operation ‘randomise’. The ‘Experiment’ package is a software extension to the statistical software R^©^. It serves to design and analyse different types of randomised trials, including cluster randomised trials and block- or matched-pair designed trials. The restricted randomisation was used to identify ten intervention and five comparison communes out of the sixteen surveyed communes balancing for four variables: HAZ, maternal education, household wealth and child dietary diversity.

Characteristics of the study groups were defined as follows:

∙ *Intervention area*: households had access to farmer field/business school training and nutrition education by the MALIS project.
∙ *Comparison area*: households had access to MALIS farmer field/business school training only.

### Agricultural intervention

Once baseline data collection and randomisation processes identifying intervention and comparison areas were completed, the MALIS project started its agricultural intervention in January 2013. The project worked through existing farmer groups and recruited participants on the basis of their interest in participating in a farmer field school on either rice or chicken or vegetables or cash-crop production. The topic varied by location following the farmers’ needs and interests. Households were eligible to participate if they had access to land. The farmer field school curriculum included field days and sessions on family nutrition. After one round of farmer field school was concluded, the group was given the opportunity to continue with one of the other topics. Farmer business schools aimed to link farmers to each other and to local markets, and were primarily offered to former farmer field school participants. In some villages, however, farmer business schools were offered instead, before or in addition to farmer field schools when the group’s interest and capacity were considered appropriate. Agricultural fairs were conducted in May/June 2014 and farmers from farmer field/farmer business schools and other farmer cooperatives from the six districts were invited. Each farmer was given a voucher to purchase items for their farm (fertiliser, seeds, tools, etc.) or kitchen equipment. The farmers were obliged to pay back 60 % of the value of the voucher to the cooperative after receiving income from harvest.

### Nutrition education programme

A training of trainers approach was used to train village health volunteers, called community nutrition promoters (CNP), in July 2013. The first round of nutrition education sessions in the project villages in the intervention area started 10 months after baseline and 6 months after the start of the agricultural intervention. National nutrition education materials were used, which were developed by the National Nutrition Programme and UNICEF in Cambodia in 2012 (Baby-Friendly Community Initiative (BFCI) flipchart). These were part of the nationwide behaviour change communication strategies that have been implemented to promote appropriate complementary feeding practices^(^ ^11^ ^)^. In addition, a facilitator guide was developed for the CNP by the MALIS project based on Trials of Improved Practices (TIP) results, that were conducted during a former FAO project in the same region. The facilitator guide assisted the CNP to structure the specific content selected for the seven sessions. The content of the sessions comprised eight key messages: continued breast-feeding, introduction of complementary foods, consistency of complementary foods, dietary diversity, feeding a sick child, responsive feeding, family nutrition and hygiene practices (Table 1). Three TIP-based cooking demonstrations were conducted at sessions 3, 4 and 5. Educational posters, soap and kitchen equipment were provided to the participants. Two sharing meetings enhanced the exchange of experience, questions, barriers and motivational factors between participants and trainers. CNP had additional sharing meetings with the other facilitators. A public announcement in each farmer field/business school village called for interested caregivers with children aged 5–18 months. In each village, a group of fifteen caregiver–child pairs was selected for nutrition education sessions. Trained CNP together with local NGO conducted seven nutrition education sessions of 2–4 h weekly or biweekly depending on the availability of the participants. Supervision was regularly performed by FAO nutrition officers and assistants.

**Table 1:** Content of nutrition education sessions*

*n*	Key messages	Content
1	Awareness of IYCF, food safety, hygiene practices	∙ Introduction ∙ Before cooking: wash your hands with clean water and soap; wash foods with clean water; wash knife and cutting surface ∙ Cover food and store utensils in a clean place ∙ Before eating: wash your hands and baby’s hands with clean water and soap ∙ Wash your hands with clean water and soap after using the toilet or cleaning the baby’s bottom
2	Continued breast-feeding, dietary diversity, food for lactating mothers	∙ Breast-feed your child on demand in addition to giving complementary foods ∙ Continue to breast-feed your child until he/she is 2 years of age or older ∙ From the age of 6 months, feed your child enriched *borbor* † made with meat or fish or egg or beans and vegetables ∙ Give your child fruits such as banana or mango or other soft fruits ∙ A lactating mother should be eating four meals/day to be healthy and produce breast milk ∙ A mother should regularly go to the health centre for check-ups ∙ In the health centre, mothers will get vitamin A capsules, Fe/folate tablets ∙ If you live in a zone with malaria, make sure you and your baby sleep under insecticide-treated bed net to prevent malaria
3	Dietary diversity, consistency	∙ Feed your child animal-source foods such as fish or meat or egg or beans every day ∙ Feed your child vegetables every day ∙ Feed your child with a separate bowl and spoon
Sharing meeting		
4	Dietary diversity, consistency, responsive feeding	∙ Peanuts provide energy and fat and are part of the body-building foods and will help children grow strong ∙ Eggs are part of the body-building foods and can be used when preparing enriched *borbor* † for your child ∙ Make mealtimes a relaxed and happy time for the child, that is clap your hands, make funny faces, demonstrate opening your own mouth very wide and say encouraging words ∙ Feed slowly and patiently, encourage your child to eat but do not force them
5	Quantity (age-appropriate), dietary diversity	∙ Gradually increase the quantity and frequency of complementary foods as your child grows older ∙ Feed your baby ripe fruits for snacks such as banana, papaya and mango
Sharing meeting		
6	Having a separate bowl for the child, feeding a sick child	∙ If the baby has diarrhoea or is vomiting, he/she should be taken to the health centre or hospital immediately to get medication such as oral rehydration solution ∙ A sick child should be given more fluids and food: breast-feed more, give more frequently thick *borbor* †, coconut water and plain clean water ∙ As the child is not feeling well, it is important to feed the child with patience and encourage him/her to eat by talking to him and helping him/her to eat
**7**	Review of key messages, graduation	

* Derived from the national nutrition education materials produced by the National Nutrition Program and UNICEF.

† *Borbor* is the Khmer word for porridge, which is traditionally made with rice.

The research team closely monitored the intervention, specifically the nutrition education sessions, but at no point actively influenced the implemention of the MALIS project’s design and process.

Nutrition education posters that were developed by the MALIS nutrition team were introduced during training and handed out to the caregivers as a reference afterwards. This facilitated dissemination of the information at the end of the first round of nutrition education sessions. The content of the posters included recipes for complementary foods, age-appropriate feeding, sanitation and hygiene, food preparation and a seasonal food availability calendar.

### Impact survey

An impact survey was conducted 2 years after baseline in the format of a census in all MALIS project villages targeted since 2012. This resulted in a total of forty-six villages (thirty-two villages in the intervention and fourteen in the comparison area). Information on children aged 0–23 months was obtained from each village in close collaboration with village chiefs and village health volunteers. An estimated number of 1172 children aged 0–23 months were eligible for the survey. Because of absence or migration of the primary caregiver (*n* 43), children being older than 23 months (*n* 40), refusals (*n* 11) or children passing away (*n* 2), 1076 caregiver–child pairs finally participated in this study.

For the analyses in this study, only children in the complementary feeding age group of 6–23 months were considered, as this study focused on the impact of nutrition education on dietary diversity. The final sample sizes comprised 743 caregiver–child-pairs at baseline and 921 at impact.

### Data collection procedure in the field

Data collection at baseline and impact followed a similar procedure. In each village, the selected primary caregivers with their children were invited to a central meeting point for participating in the survey. The children’s ages were verified at this point by cross-checking the birth dates indicated on village lists with the vaccination cards or birth certificates. If there was no information on the child’s age, the age was estimated using a local events calendar and later dated to the 15th of the named month.

Semi-structured questionnaires, which included a household, child and caregiver section, were administered via face-to-face interviews with the primary caregiver of the under 2-year-old child in the selected household. Data collected included socio-economic and demographic information on the household, as well as household and child dietary diversity scores (CDDS) based on 24-h recall, and child’s 7-d food frequency (FFQ). In addition, feeding and caring practices including hygiene were assessed. In addition, episodes of fever, diarrhoea and acute respiratory infections as perceived by the caregiver were recorded for the 2 weeks preceding the interview. All data collection tools were pre-tested in the field. The supervisors regularly observed interviews and anthropometric measurements filling in a quality control form. If needed, refresher training was provided. An immediate questionnaire translation was carried out after each interview and was cross-checked by a native speaker and the German research team. At impact, enumerators were blind to group assignment.

### Anthropometry

Anthropometric measurements were taken of the mother and child with standardised equipment from Seca (Seca GmbH & Co. KG): digital flat weighing scales with mother–child function (Seca 874, capacity 200 kg; SECA; kg to two decimal points), length boards (Seca 417, measurement range 10–100 cm; SECA) and stadiometers (Seca 213, measuring range 20–205 cm; SECA). Mothers’ heights and weights were collected as well as the children’s lengths and weights following a standardised protocol. Height/length and weight were assessed to the nearest 0·1 cm and 0·1 kg, respectively^(^ ^12^ ^)^. All measurements were taken twice. The maximum tolerated difference between the two measurements was 1·0 cm for height/length and 0·5 kg for weight at baseline^(^ ^12^ ^)^ and 0·7 cm for height/length and 0·15 kg at impact^(^ ^13^ ^)^. The mean of both measurements was used for the final analysis. HAZ, weight-for-age *Z*-scores (WAZ) and weight-for-height *Z*-scores (WHZ) were created using SPSS Macro (adopted) from the World Health Organization^(^ ^14^ ^)^.

### Wealth index

Socio-economic data were used to develop an adapted local wealth index based on the results of a principal component analysis. Variables included in the wealth index were housing, people per sleeping room, floor composition, type of sanitation and drinking water source. In addition, ownership of land and certain assets (e.g. radio, television, mobile and non-mobile phone, wardrobe, sewing machine or loom, CD/DVD player, generator/battery/solar panel, watch, bicycle, motorcycle, motorcycle-cart, car/truck/van, boat, ox-/horse-cart and hand-tractor) were considered^(^ ^15^ ^,^ ^16^ ^)^. For this analysis, a wealth index created together for baseline and impact was used.

### Indicators for infant and young child feeding

Feeding practices were assessed using the following WHO IYCF indicators for children aged 6–23 months: continued breast-feeding, introduction of solid, semi-solid and soft foods, minimum dietary diversity (MDD), minimum meal frequency (MMF) and minimum acceptable diet (MAD)^(^ ^17^ ^,^ ^18^ ^)^. These indicators look at the percentage of children meeting the recommended criteria. CDDS was calculated using a seven-food-group score, reflecting the consumption of seven different food groups in the past 24 h: grains, roots and white tubers, legumes, nuts and seeds, dairy products, flesh foods (meat, poultry, fish and offal), eggs, pro-vitamin A-rich foods (yellow and orange-fleshed roots and tubers, orange-fleshed fruits and dark green leafy vegetables) and other fruits and vegetables^(^ ^18^ ^)^. This score assesses whether or not the child had eaten food from a certain food group, and not the quantity consumed. In addition, food consumption in the past 7 d was determined by a FFQ. A child feeding index was created assessing five different IYCF components compiled into one index, adjusted for child’s age: continued breast-feeding, no bottle-feeding, dietary diversity, meal frequency and food frequency^(^ ^19^ ^)^.

### Statistical analysis

Double entry of all data was performed using EpiData (version 3.1). Analyses were carried out using SPSS (SPSS Statistics version 20.0.0.2; IBM). Before testing for associations between different indicators, data were tested for intra-class correlations (ICC) at the village level using the procedure MIXED in SPSS. All statistical models were accounted for ICC. As sample sizes were large, the Wald’s test was interpreted. *t* Tests were used to determine differences between groups. Pearson’s *R* is reported as a standardised measure of effect size. To take differences at baseline into account, as well as the impact of other nutrition education programmes on both groups, difference-in-differences (DiD) models were applied using linear regression^(^ ^20^ ^)^. A DiD model is often applied in quasi-experimental studies with repeated cross-sectional data with intervention and comparison groups. The idea is to control the intervention effect for baseline differences and for time effects (general development without intervention), just as a repeated-measures ANOVA would do for longitudinal data. In the linear model, the formula for the DiD model is as follows:
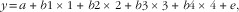
where *y*=dependent variable, *a*=constant, *b*1…*b*4=non-standardised regression coefficients, *b*4=vector of coefficients, *x*1=dummy for time with baseline=0 and follow up=1, *x*2=dummy for group with 0=comparison group and 1=intervention group, *x*3=*x*1×2 with 1 for intervention group at follow up and 0=otherwise, *x*4=vector of covariates in the model and *e*=estimated error term.

Dependent variables were either CDDS or single food groups or HAZ. Age of child, maternal education and household wealth were included as covariates in all models, and maternal height and sex of the child were included only in the analysis with HAZ. All covariates were grand mean centred beforehand. Reported coefficients were unstandardised. Associations between HAZ and CDDS were assessed using partial correlation with and without control variables. Linear probability models with robust standard errors^(^ ^21^ ^)^ were calculated to determine differences of particular food groups. The term ‘linear probability model’ refers to a linear regression model. ‘Probability’ refers to the interpretation of the estimated dependent variable – that is, the probability that a food group is consumed^(^ ^20^ ^)^. In SPSS, the heteroscedasticity-consistent standard error estimators procedure by Hayes & Cai was used^(^ ^22^ ^)^.

## Results

A total of 1664 data sets were used for analysis consisting of 743 and 921 caregiver–child pairs from baseline and impact surveys, respectively. Children’s age ranged from 6 to 23 months with a mean age of 13·5 (sd 5·2) months at baseline and 14·2 (sd 5·2) months at impact. Main household and child characteristics are presented in Table 2. Between baseline and impact, the ownership of home gardens and animals decreased in both groups. The majority of households had access to arable land with a mean size increasing towards impact, ranging from 1·7 to 2·3 ha at baseline and from 2·4 to 2·5 ha at impact. The access to improved sanitation facilities was higher at impact compared with baseline in both groups. Average maternal education was higher and households had a more diverse diet at impact in both groups in comparison with baseline. Both groups had a mean household food insecurity access scale score of 6 (min, max: 0–19).

**Table 2:** Main household and child characteristics† (Mean values and standard deviations)

	Baseline					Impact				
Indicators	Comparison (*n* 233)		Intervention (*n* 510)		*P* *	Comparison (*n* 397)		Intervention (*n* 524)		*P* *
Household										
% Access to arable land	89·7		93·5		0·069	91·7		94·5		0·098
Size of arable land (ha)					<0·001					0·651
Mean	1·72		2·26			2·36		2·48		
sd	1·33		2·03			5·21		2·81		
% Home garden	76·0		67·5		0·019	56·4		58·8		0·447
% Access to fruits	86·7		81·4		0·073	87·9		88·9		0·640
% Ownership of animals	88·4		92·5		0·064	83·1		91·6		<0·001
% Access to improved sanitation facilities	19·7		19·0		0·817	28·5		27·1		0·664
% Access to a protected source of drinking water	85·8		87·6		0·495	884		86·3		0·326
Education (years)										
Respondent					0·871					0·511
Mean	3·4		3·5			4·4		4·3		
sd	3·1		3·1			3·5		3·5		
Household head (if not respondent)					0·339					0·011
Mean	4·0		4·3			5·3		4·5		
sd	3·9		3·7			3·9		3·9		
Household dietary diversity score (min–max: 2–12)					0·099					0·154
Mean	7·0		6·8			7·6		7·8		
sd	1·6		1·7			1·7		1·7		
Wealth index score					0·906					0·944
Mean	−0·6		−0·6			0·5		0·5		
sd	2·8		3·1			3·3		3·2		
Wealth index quintiles										
% Lowest	25·0		29·2			14·1		13·7		
% Second	20·7		16·1			20·4		21·2		
% Middle	22·0		20·8			20·4		19·3		
% Fourth	19·4		19·2			21·7		19·8		
% Highest	12·9		14·7			23·4		26·0		
Child										
Age (months)					0·406					0·396
Mean	13·8		13·5			14·4		14·1		
sd	5·3		5·1			5·4		5·2		
% Sex (female)	48·5		43·1		0·173	46·9		49·0		0·486
% Delivery by professional health staff	82·0		76·7		0·103	94·4		91·0		0·049
% Vitamin A supplement (past 6 months)	83·6		83·2		0·878	62·4		59·1		0·297
% Deworming tablet (past 6 months)	47·4		40·4		0·075	34·0		30·9		0·330
Illness past 2 weeks (as perceived by respondent)										
% Fever	69·1		67·1		0·581	76·8		71·2		0·052
% Diarrhoea	41·6		36·9		0·222	26·2		27·9		0·558
% ARI	22·0		17·3		0·279	27·2		22·3		0·813
WHO indicators (% achieved)										
% Introduction of semi-solid/soft foods (6–8 months)	95·9 (*n* 49)		91·7 (*n* 96)		0·339	92·6 (*n* 68)		88·1 (*n* 84)		0·349
% Continued breast-feeding (12–15 months)	92·2 (*n* 51)		92·6 (*n* 122)		0·916	85·5 (*n* 76)		89·1 (*n* 117)		0·377
% Continued breast-feeding (20–23 months)	44·9 (*n* 49)		50·0 (*n* 96)		0·561	34·3 (*n* 99)		31·9 (*n* 116)		0·704
% Minimum dietary diversity (6–23 months)	50·2 (*n* 233)		44·3 (*n* 508)		0·133	55·9 (*n* 398)		64·9 (*n* 524)		0·006
% Minimum meal frequency (6–23 months)	69·0 (*n* 232)		66·5 (*n* 508)		0·513	83·4 (*n* 398)		86·4 (*n* 523)		0·204
% Minimum acceptable diet (6–23 months)	33·2 (*n* 232)		27·2 (*n* 508)		0·118	36·2 (*n* 398)		45·5 (*n* 523)		0·004
Child feeding index (min–max: 0–10)					0·309					0·107
Mean	6·8		6·7			7·2		7·3		
sd	1·6		1·7			1·7		1·6		
Nutritional status										
Height-for-age *Z*-scores					0·690					0·400
Mean	−1·24		−1·27			−1·27		−1·33		
sd	1·03		1·17			1·09		1·09		
Weight-for-height *Z*-scores					0·857					0·914
Mean	−0·75		−0·77			−0·63		−0·63		
sd	1·01		1·04			0·98		0·99		
Weight-for-age *Z*-scores					0·643					0·730
Mean	−1·19		−1·23			−1·13		−1·15		
sd	0·99		1·08			0·97		0·99		

ARI, acute respiratory infections.

* *P* values for comparison between groups, separately for the surveys and not corrected for multiple comparison.

† *t* Test for data where mean values are reported, Pearson’s *χ* ^2^ test for data where percentages are reported.

The number of children receiving vitamin A supplements and deworming tablets was lower at impact than at baseline. Prevalence of diarrhoea decreased in both groups between baseline and impact.

### Coverage of the FAO project

**Fig. 2:**
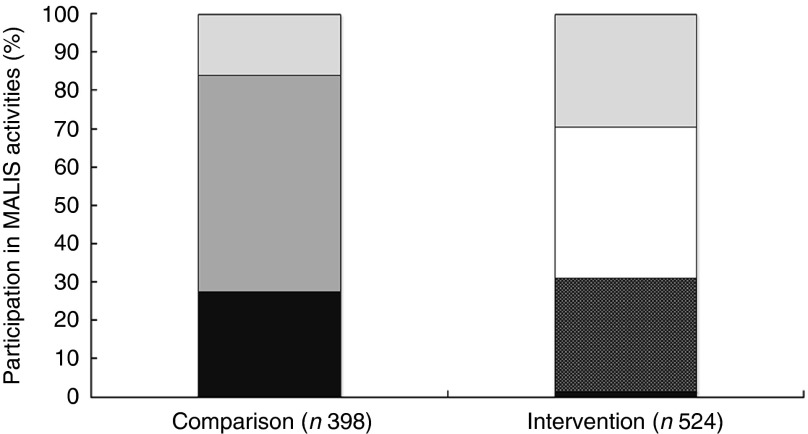
Participation in FAO activities at impact. Participation in any other nutrition education or food security activities is not presented in this figure. MALIS, improving market linkages for smallholder farmers; 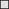, don’t know; 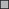, no participation in any MALIS activity at impact survey; 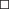, nutrition education; 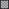, nutrition education+farmer field/business school; 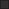, farmer field/business school.

Overall, 79 % of the households in the intervention group and 25 % of the comparison group stated that they had participated in some kind of nutrition education programme offered in the project region. Participation in the MALIS nutrition education was assessed by confirming the presence of MALIS educational posters in the respondent’s house, proving that 69 % of the households in the intervention area had actually participated in the 12 months before the survey. Participation in a farmer field/business school was 32 % in the intervention and 27 % in the comparison group at the time of the impact survey. The overlap between FAO agriculture intervention and nutrition education was 30 % in the intervention group (Fig. 2).

### Infant and young child feeding

Continued breast-feeding at 12–15 months and 20–23 months decreased in both groups (Table 2). In contrast, a higher number of children achieved MDD, MMF and MAD in both groups at impact.

**Fig. 3:**
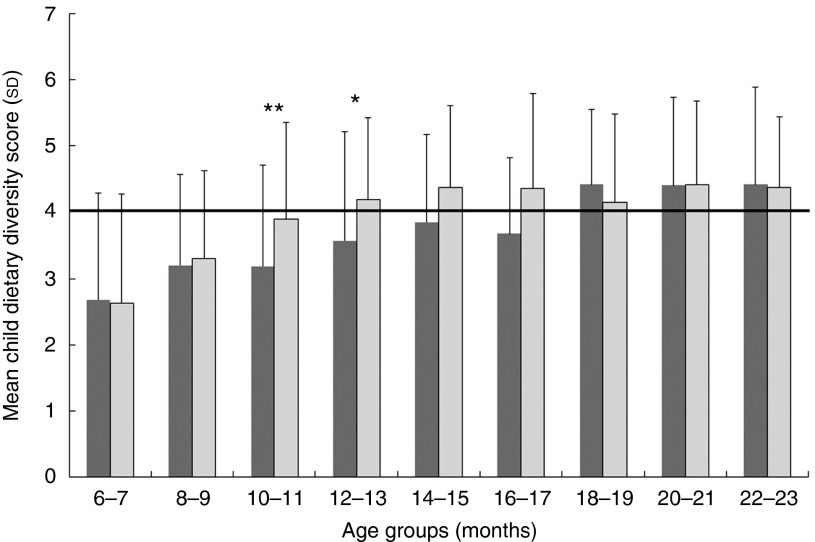
Mean child dietary diversity scores (+1 sd) of children in the intervention and comparison groups by 2-month age groups. Number per age group (months) comparison/intervention: 6–7=50/59; 8–9=43/58; 10–11=57/83; 12–13=37/69; 14–15=39/52; 16–17=21/42; 18–19=50/44; 20–21=59/58; 22–23=38/55. Group differences with independent sample *t* test: ** *P*<0·01, * *P*<0·05. WHO recommended minimum number of food groups to be consumed in 1 d. 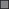, Comparison (*n* 394); 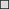, intervention (*n* 520).

The consumption of all food groups increased in the intervention group, whereas the consumption of pro-vitamin A-rich foods and animal source foods (ASF) decreased in the comparison group (Table 3). The overall consumption of ASF and sugary foods and processed snacks was high. The mean CDDS increased by 0·2 score points in the comparison group and by 0·6 in the intervention group. Mean CDDS increased with age in both groups (Fig. 3). From 10 to 18 months of age, children in the intervention group showed higher mean CDDS than children of the same age in the comparison group. However, this was only significant for 10- to 11-month-olds (*R* 0·23, *P*=0·007) and 12- to 13-month-olds (*R* 0·21, *P*=0·03).

**Table 3:** Descriptive characteristics of food consumption (24-h recall) (Mean values and standard deviations)

Food group	Baseline				Impact			
(% of children aged 6–23 months consumed)	Comparison (*n* 233)		Intervention (*n* 508)		Comparison (*n* 397)		Intervention (*n* 524)	
Grains, roots, white tubers*	97·4		95·3		96·5		97·5	
Flesh foods*	79·4		73·9		76·3		77·7	
Other fruits and vegetables*	56·7		48·2		57·9		65·1	
Pro-vitamin A-rich foods*	51·1		43·3		47·6		55·7	
Eggs*	29·6		32·7		36·0		46·0	
Legumes, nuts, seeds*	21·9		16·3		34·0		35·1	
Dairy products*	8·6		11·6		21·9		16·4	
Animal source foods	82·8		81·8		82·6		89·5	
Dark green leafy vegetables	41·2		29·4		37·5		46·2	
Pro-vitamin A-rich roots and tubers	24·0		23·1		20·4		38·2	
Pro-vitamin A-rich fruits	6·0		4·9		6·3		7·1	
Fats and oils	40·8		33·3		57·4		58·8	
Sugary foods and crisps	60·1		58·4		75·8		70·2	
CDDS (0–7)								
Mean	3·5		3·2		3·7		3·9	
sd	1·6		1·5		1·5		1·5	

CDDS, child dietary diversity score.

* One out of the seven food groups the CDDS consists of.

### Nutritional status

**Fig. 4:**
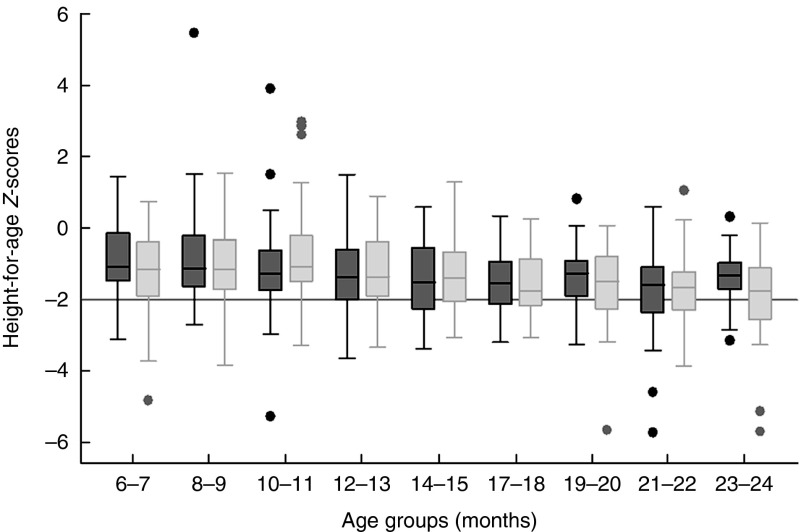
Mean height-for-age *Z*-scores (±1 sd) of children in the intervention and comparison groups by 2-month age group at impact. Number per age group (months) comparison (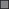)/intervention (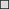): 6–7=51/58; 8–9=43/58; 10–11=57/82; 12–13=37/69; 14–15=39/52; 16–17=20/42; 18–19=49/44; 20–21=59/58; 22–23=38/55.

Mean HAZ scores at baseline were at −1·24 (sd 1·03) for the comparison group and at −1·27 (sd 1·17) for the intervention group. At impact, mean HAZ scores were −1·25 (sd 1·12) and −1·32 (sd 1·12) for comparison and intervention, respectively. Fig. 4 shows that the vast majority of infants and young children were growing well or in the lower normal range. The median HAZ did not vary much between intervention and comparison groups. Average stunting prevalence at impact was 23·5 and 24·7 % in the comparison and intervention groups, respectively.

### Association between dietary diversity and nutritional status

In a partial correlation model, HAZ scores were weakly but not significantly correlated with CDDS when including covariates: *R* 0·05, *P*=0·06.

### Effects of the intervention on children’s diets and nutritional status

**Fig. 5:**
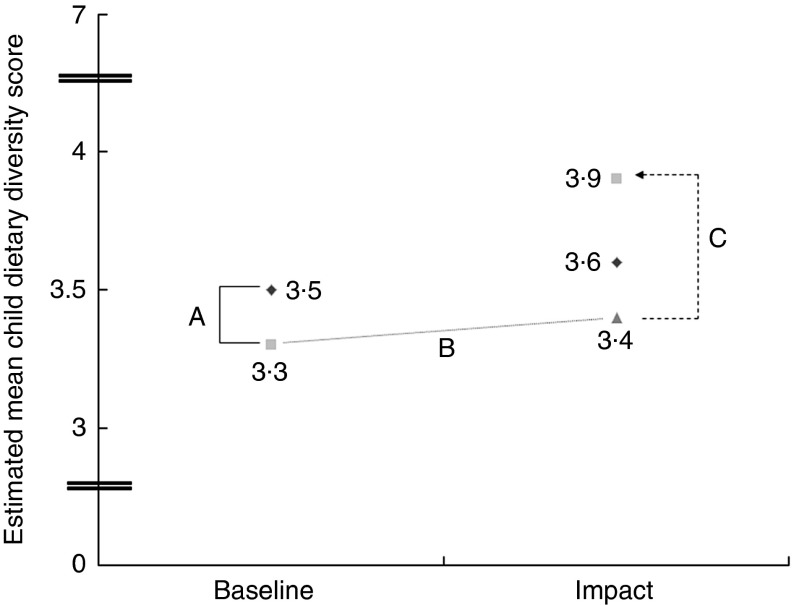
Differences in estimated mean child dietary diversity (differences-in-difference (DiD) model). A, differences between comparison and intervention at baseline=−0·22, *P*=0·048; B, hypothetical development of intervention group without intervention (DiD assumption); C, treatment effect=0·49, *P*=0·001; 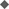, comparison; 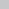, intervention; 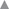, intervention (counterfactual).

At impact, the estimated mean CDDS was 3·6 and 3·9 in the comparison and intervention groups, respectively (Fig. 5). Improvements in CDDS were reflected in a significant positive treatment effect (*B*=0·52, se(*B*)=0·18; 95 % CI 0·17, 0·87, *P*=0·005) controlled for differences at baseline and between groups and covariates; thus, the intervention’s CDDS improved by 0·52 food groups of the mean. An increased CDDS was mainly attributed to increased consumption of pro-vitamin A-rich foods and other fruits and vegetables. The intervention showed a negative significant treatment effect on consumption of dairy products. Treatment effects on the consumption of legumes, nuts and seeds, flesh foods and eggs were positive but not significant (Table 4). If all ASF were combined into one food group, a DiD model including age of child, maternal education and wealth showed a positive treatment effect (*B*=0·09, se(*B*)=0·04; 95 % CI 0·008, 0·17, *P*=0·030). Age of child and maternal education as covariates were significantly associated with the model.

**Table 4:** Treatment effects on children’s food consumption (Linear probability models with robust standard errors)

Food group (24-h recall)	Treat effect	se (*B*)	*P*	95 % CI
Legumes, nuts, seeds	0·08	0·06	0·159	−0·03, 0·189
Dairy products	−0·09	0·05	0·073	−0·18, 0·01
Flesh foods	0·08	0·04	0·079	−0·01, 0·17
Eggs	0·08	0·05	0·107	−0·18, 0·17
Pro-vitamin A rich foods	0·16	0·05	0·003	0·06, 0·26
Other fruits and vegetables	0·17	0·06	0·003	0·06, 0·28

On the basis of the 7-d food frequency, significant treatment effects on the consumption of specific foods were determined by a DiD model including age of child, wealth and maternal education: fish (*B*=0·73, se(*B*)=0·36; 95 % CI 0·02, 1·44, *P*=0·05), pro-vitamin A-rich roots and tubers (*B*=1·11, se(*B*)=0·25; 95 % CI 0·62, 1·60, *P*<0·001) and dark green leafy vegetables (*B*=1·15, se(*B*)=0·33; 95 % CI 0·51, 1·80, *P*=0·001). Maternal education as a covariate was significantly associated with increased consumption of pro-vitamin A-rich roots and tubers (*P*=0·017) as well as dark green leafy vegetables (*P*=0·01), whereas age was significantly associated with the increased consumption of dark green leafy vegetables (*P*<0·001) only. Wealth was significantly associated with increased consumption of fish (*P*=0·003) and pro-vitamin A-rich roots and tubers (*P*<0·001).

No significant treatment effects of the nutrition education intervention on HAZ, WAZ and WHZ were observed.

### Determinants of child dietary diversity

Wealth and age of the child were determinants of child dietary diversity at baseline (wealth: *B*=0·08, se(*B*)=0·02, *β*=0·17, *P*<0·001) and impact (wealth: *B*=0·09, se(*B*)=0·02, *β*=0·19, *P*<0·001). The older the child and/or the wealthier the household, the more diverse the child’s diet was. Maternal education was positively associated with child dietary diversity in the same model at impact only (*B*=0·06, se(*B*)=0·01, *β*=0·15, *P*<0·001). Household dietary diversity was significantly associated with child dietary diversity at impact (*B*=0·41, se(*B*)=0·02, *β*=0·47, *P*=0·011) in a model including group, age of child, maternal education and wealth as confounders.

## Discussion

In this study, we could show that the nutrition education intervention embedded in an agriculture project led to significant improvements in the quality of children’s diet. However, the mean diversity of children’s diet remained just below the minimum level of four out of seven food groups as recommended for young children by the World Health Organization^(^ ^23^ ^)^. Other studies with similar nutrition education messages, but different approaches, also reported improvements in dietary diversity^(^ ^24^ ^,^ ^25^ ^)^. Nutrition education delivered through home-visit counselling improved dietary diversity in India and overall energy and nutrient intake in Malawi^(^ ^26^ ^–^ ^29^ ^)^. Nutrition education through intensive training given to small groups resulted in a positive impact on caregiver’s nutrition and health knowledge and practices in Indonesia^(^ ^30^ ^)^.

The best practices have been summarised in several reviews, but the scientific evidence on nutrition education projects in development cooperation and their impact on growth is limited. In contrast to other studies, proven changes in feeding practices of the respondents were not reflected in changes of HAZ scores in our study. In China, *Z*-scores of children, whose caregivers participated in a nutrition education intervention, started to increase after 10–11 months of education^(^ ^31^ ^)^. In Peru^(^ ^32^ ^)^, improved IYCF practices following nutrition education impacted on child’s growth after a period of 18 months of counselling by health sector staff, including regular home visits for interview and anthropometric measurements. In this study, the evaluated nutrition education was only carried out for 12 months, and was thus probably too short to observe an impact on HAZ. Another explanation might be the limited exposure of behaviour change communication messages (six sessions).

A study from Pakistan showed that an education programme on complementary feeding had a direct positive impact on linear growth of infants^(^ ^33^ ^)^. A total of four home visits delivered educational messages on complementary feeding every 10 weeks over a period of 10 months. An analysis of the Cambodian Demographic and Health Survey 2005 data by Darapheak *et al*.^(^ ^34^ ^)^ suggested a positive impact of ASF consumption on the reduction of stunting. In this study, the percentage of children who consumed foods from the food group ‘flesh foods’ (meat, poultry, offal and fish) was high (77 %), but this was mainly attributed to high fish consumption rather than meat, poultry or offal. Fish is a part of the daily Cambodian diet and commonly available and affordable in rural areas, especially in the rainy season. Best sources of Fe, however, are meat and offal, which are more expensive and less consumed by poorer, rural households.

Other studies that reported a positive impact on HAZ scores were characterised by their impact on increased intake of protein-rich foods such as eggs, legumes, nuts and seeds^(^ ^31^ ^,^ ^32^ ^,^ ^34^ ^)^. The lack of a significant impact on HAZ scores in this study might also be attributed to the amount consumed by a child during a meal, which could be limited by the high level of consumption of processed snacks. Where consumption of snacks is high and the caregiver’s knowledge on responsive feeding behaviours is poor, meal patterns are less structured and children do not display hunger^(^ ^35^ ^,^ ^36^ ^)^. In addition, during this window of opportunity, complementary feeding only plays a contributory role and its impact on HAZ should be observed in addition to growth before birth and during the first 6 months of life.

As known from previous research, a set of good IYCF practices is associated with the nutritional status of the children^(^ ^19^ ^,^ ^37^ ^)^. Although food-based approaches are often questioned, their applicability remains obvious, especially in remote and poverty-affected areas^(^ ^38^ ^,^ ^39^ ^)^. Nutrition education alone is able to improve caregivers’ awareness of the importance of complementary foods impacting on IYCF practices and subsequently enhancing the quality of children’s diets.

The nutrition education carried out by the FAO project and local partners increased the intake of micronutrients and bioactive plant components, particularly through increased consumption of fruits and vegetables. In general, respondents of the intervention group had increased access to fruits, which could be due to raised awareness of the availability of these foods. This, furthermore, led to increased utilisation for young children. In general, CDDS was strongly correlated with maternal education and age of the child. The latter was also found to be a determinant of CDDS in Tanzania^(^ ^40^ ^)^.

In the present study, the mean CDDS of the 6- to-11-month- olds was below the WHO recommendation, which leads to the recommendation to address lactating mothers with nutrition education programmes. Children from birth up to 18 months in Peru^(^ ^32^ ^)^ and 12 months of age in India^(^ ^27^ ^)^ were followed-up with home-visit nutrition education resulting in improved growth. Enrolment of mothers when children are still <6 months of age could strengthen IYCF practices, also with respect to breast-feeding. Despite the fact that the continuation of breast-feeding was part of the FAO’s nutrition education curriculum, its messages did not seem to impact on caregivers’ behaviour.

### Strengths and limitations of the study

There are a number of aspects reducing the effect of nutrition education on HAZ in the setting of this study: first, the majority of children studied were not stunted but growing in the lower normal range; second, the overlap between the food security and nutrition education intervention was low with 30 %; third, the presence of nutrition education activities in the project’s comparison area; and, finally, the nutrition education was performed for a shorter period than that initially planned.

The selection criteria and timing for the interventions reduced the number of households with children aged 5–18 months eligible to participate in both interventions. In addition, a high number of different actors and an unexpectedly high level of staff turnover disrupted the communication between agriculture and nutrition staff. However, it indicates that agriculture projects need to be targeted towards farm families with young children.

As this was an applied research study, the research team also had no influence on whether nutrition education was carried out by other projects or within other programmes in the project area. Thus, households may have had access to information on IYCF messages and cooking demonstrations carried out by health centre staff and NGO also using government materials. As far as possible, these activities were mapped and households’ participation in any nutrition activity was assessed. Contradictory messages could be excluded, and the exposure was similar for intervention and comparison groups. None of the other observed projects used a participatory village group-teaching approach comparable with the MALIS project.

Although ownership of animals, size of land and household dietary diversity increased, this study could not demonstrate a clear, evidence-based linkage that the project’s agricultural activities contributed to these results. For future programmes, it is recommended to target the same households with both interventions at the same time to reduce food insecurity, while enhancing improved IYCF knowledge and practices. However, an earlier start of the agricultural intervention to improve households’ food security status first as intended by this project could also be a solution and create a basis for utilisation of improved knowledge. To sustainably impact on an improved nutritional status of children, the collaboration between the agriculture and the health sector needs to be strengthened through joint efforts and programmes. Qualitative data from this research, not shown here, indicate that through inclusion of nutrition education in an agriculture programme and involvement of husbands and grandparents, overall sustainable improvements could be achieved by improved nutrition knowledge as well as access, availability and utilisation of nutritious, diverse foods.

To the knowledge of the authors, only a few applied nutrition research studies, where the researchers have not designed the intervention, have shown community effects of a project to date. The necessity and benefit of such studies to scientifically evaluate such natural situations and to help in improving future programmes – ideally carried out by the government – are obvious^(^ ^26^ ^)^. As this research was attached to a development project implemented by the FAO, the researchers had no influence on NGO activities in the project region. In addition, the activities of the government’s BFCI rolled out through health centres in the project region were beyond the researchers’ control.

### Conclusion

Although the study is limited because of the named reasons, it shows the need for assessing the implementation of nutrition education to better understand the natural situations and improve coordination and overall design of such projects and programmes.

A 2–3-month nutrition education programme, carried out through government and village health volunteers as well as NGO, addressing caregivers with a child between 5 and 18 months of age, improved practices on diversification of children’s diet. As no impact on average HAZ scores could be demonstrated, we suggest that nutrition education should emphasise more on consumption of ASF and other protein sources. In addition, nutrition education in the community should be carried out through trained government and community members and include peers as trainers. It is recommended to include sessions on family nutrition in the curriculum and emphasise the continuation of breast-feeding.

To successfully combine agriculture interventions and nutrition education, the overlap between the interventions must be considered. Addressing food security and IYCF practices at the same time raises awareness and creates effective linkages between food production and nutrition.
